# Supplementary material for: Wearable Natural Rubber Latex Gloves with Curcumin for Torn Glove Detection in Clinical Settings
Source: Polymers (Basel). 2022 Jul 28;14(15):3048. doi: 10.3390/polym14153048 (PMC9370823; doi:10.3390/polym14153048)
Supplement: Supplementary file 1 [file polymers-14-03048-s001.zip › polymers-1827985-supplementary.pdf]

Supporting Information

Wearable Natural Rubber Latex Gloves with Curcumin for Torn Glove Detection in Clinical Settings

Norfatirah Muhamad Sarih[1], Nuur Syuhada Dzulkafly[1], Simon Maher[2] and Azura A. Rashid[1,*]

[1] School of Materials and Mineral Resources Engineering, Universiti Sains Malaysia, Engineering Campus, Nibong Tebal 14300, Penang, Malaysia; fatirahsarih@usm.my (N.M.S.); dn.syuhada@student.usm.my (N.S.D.)

[2] Department of Electrical Engineering and Electronics, University of Liverpool, Liverpool L69 3GJ, UK;
s.maher@liverpool.ac.uk (S.M.)

* Correspondence: srazura@usm.my (A.A.R.)

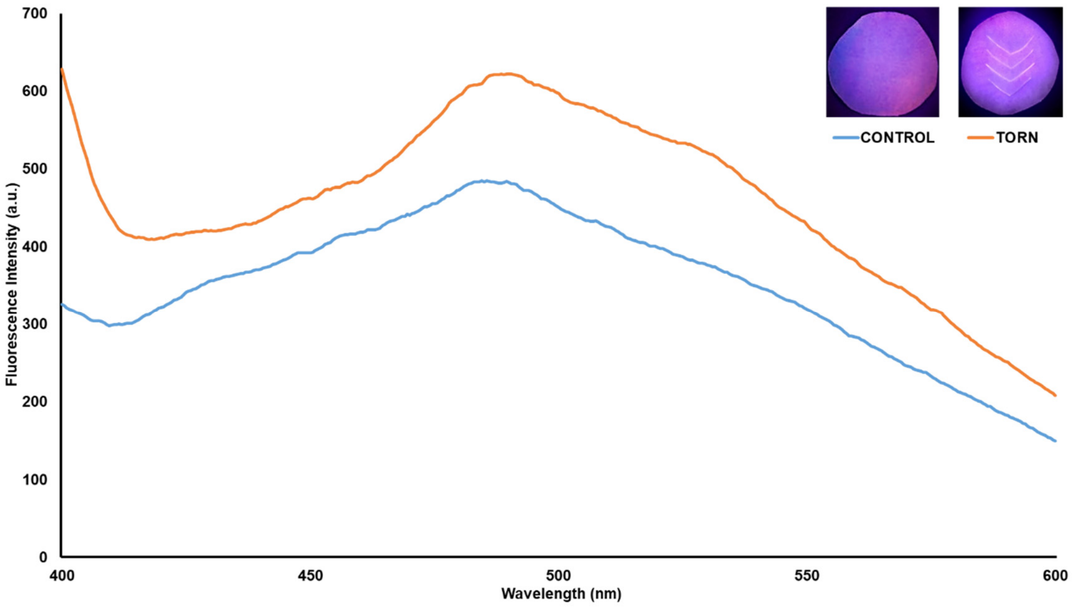

Figure S1. Fluorescence emission spectrum from **LC1** sample. (inset: NRL-Cur films under UV lamp at 380 nm)

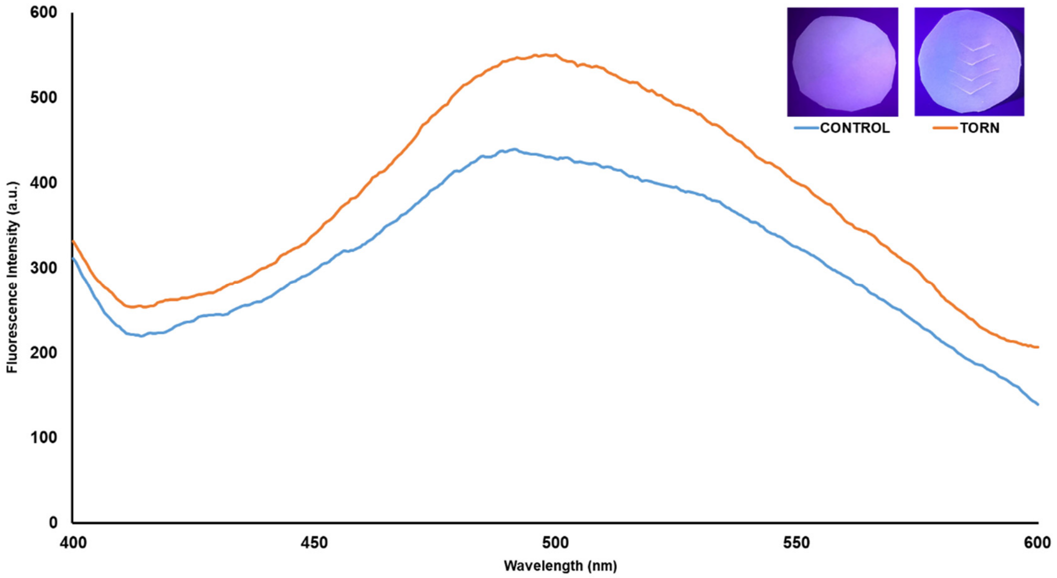

Figure S2. Fluorescence emission spectrum from **LC2** sample.(inset: **LC** films under UV lamp at 380 nm)

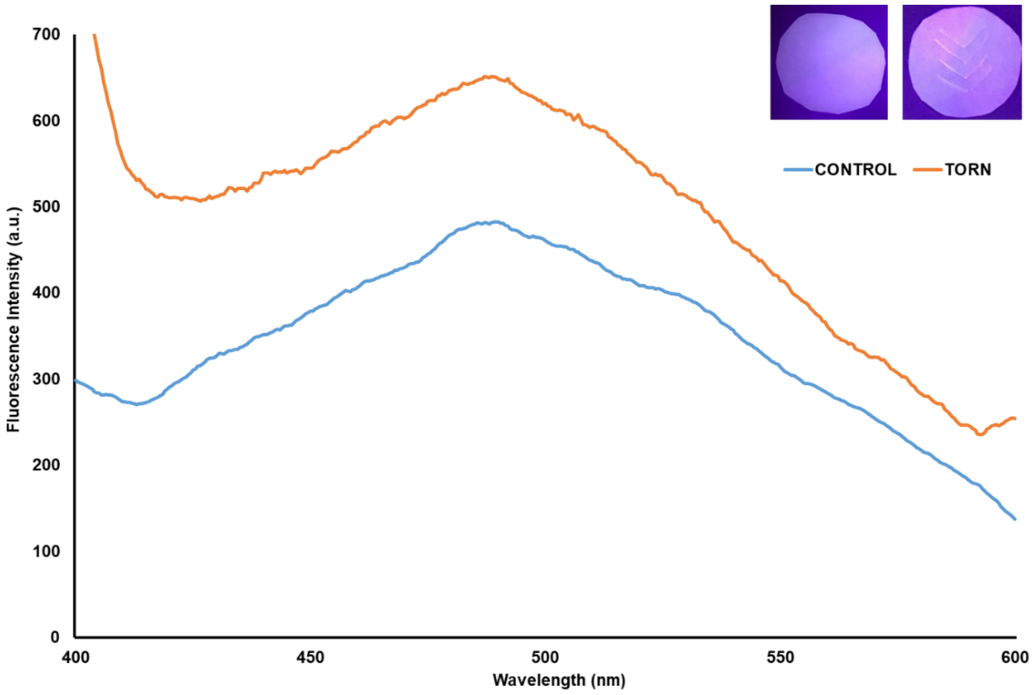

Figure S3. Fluorescence emission spectrum from **LC3** sample. (inset: NRL-Cur films under UV lamp at 380 nm)

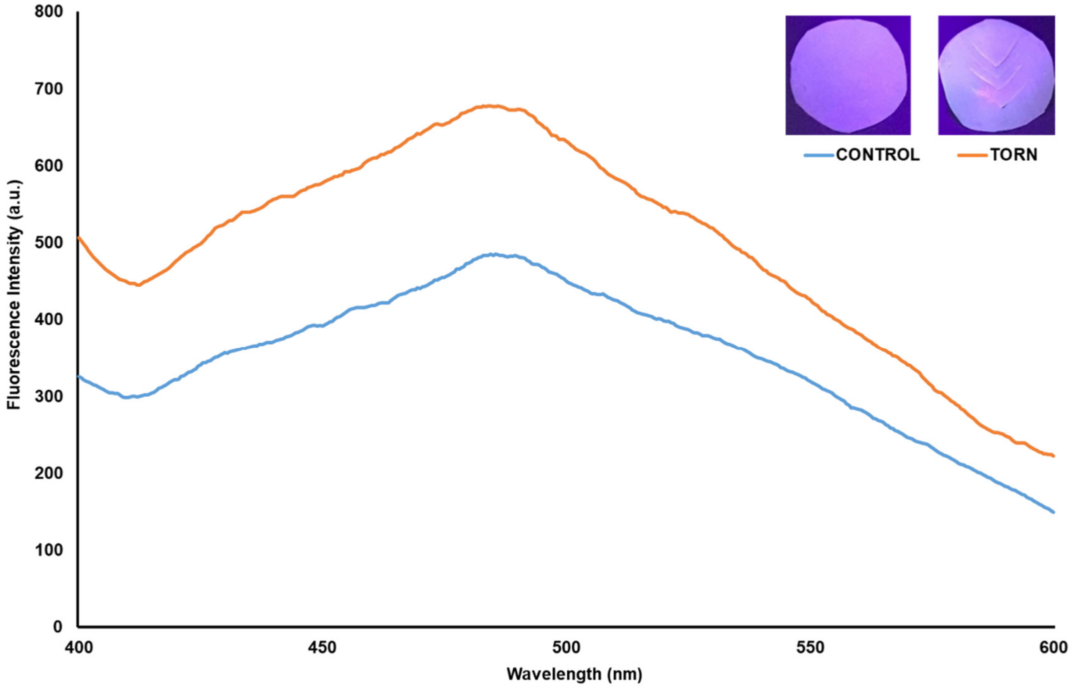

Figure S4. Fluorescence emission from **LC4** sample. (inset: NRL-Cur films under UV lamp at 380 nm)

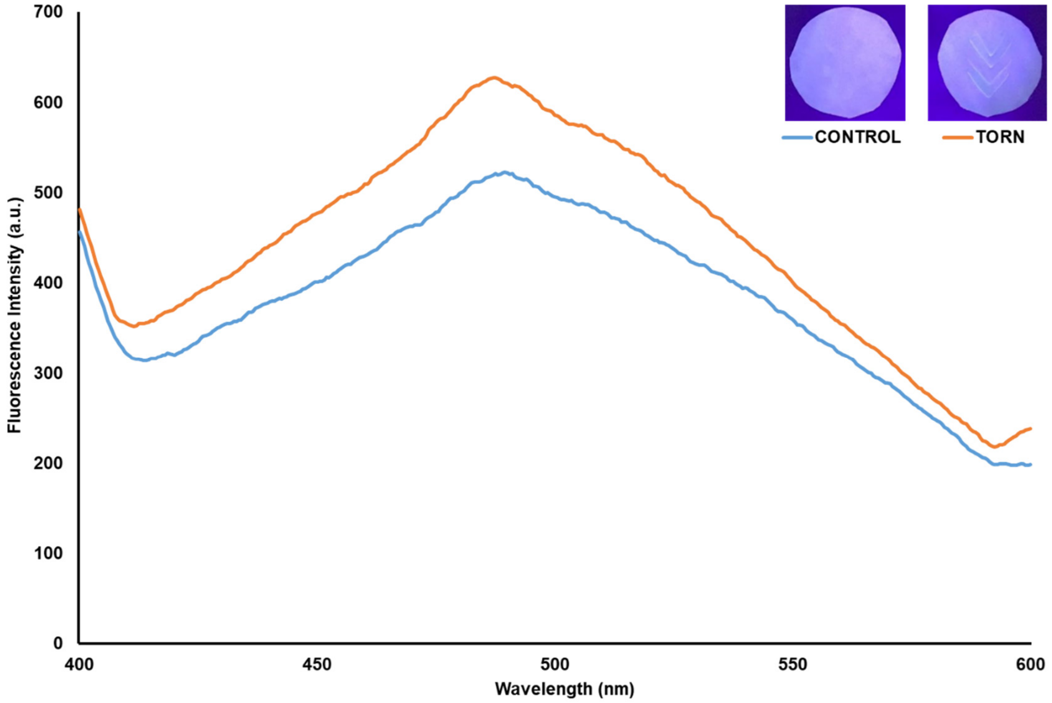

Figure S5. Fluorescence emission spectrum from **LC5** sample. (inset: NRL-Cur films under UV lamp at 380 nm)

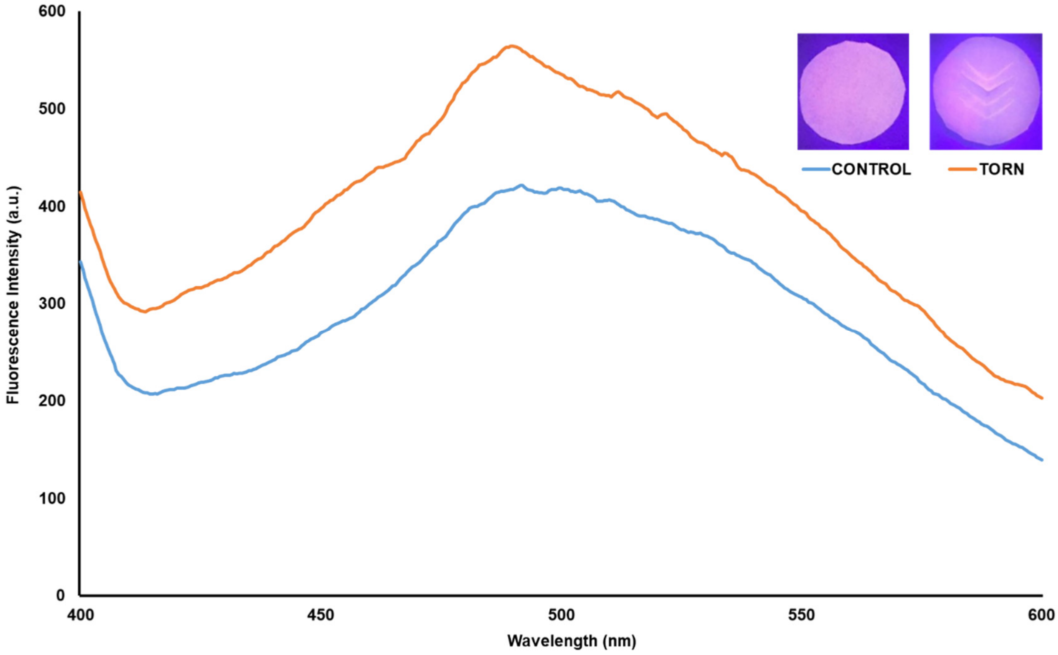

Figure S6. Fluorescence emission spectrum from **LC6** sample. (inset: NRL-Cur films under UV lamp at 380 nm)

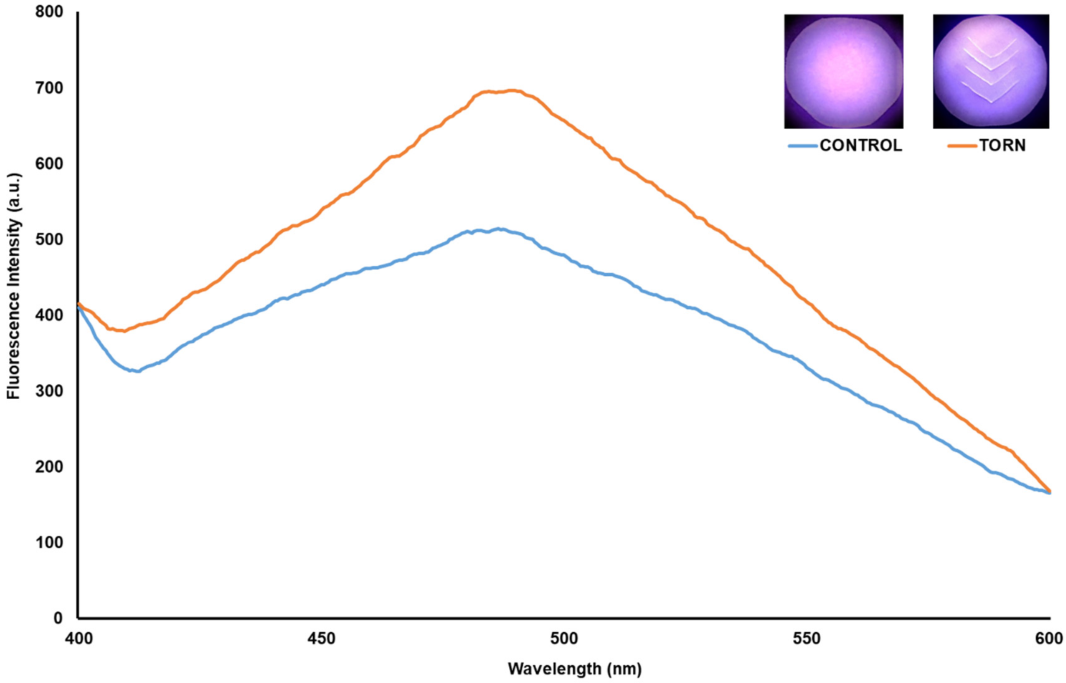

Figure S7. Fluorescence emission spectrum from **LC7** sample. (inset: NRL-Cur films under UV lamp at 380 nm)

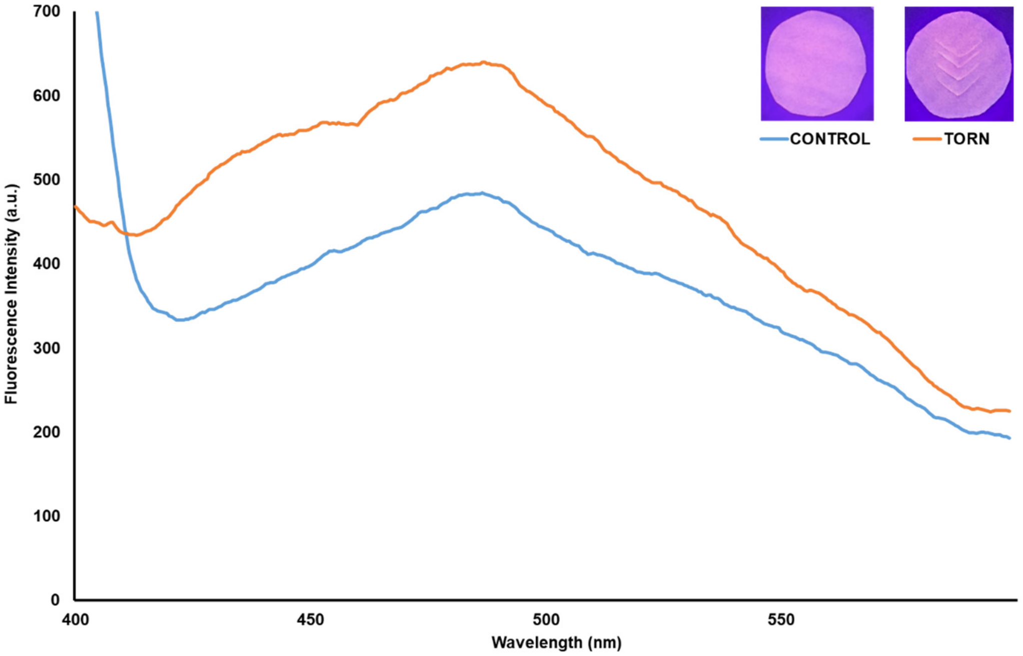

Figure S8. Fluorescence emission spectrum from **LC8** sample. (inset: NRL-Cur films under UV lamp at 380 nm)

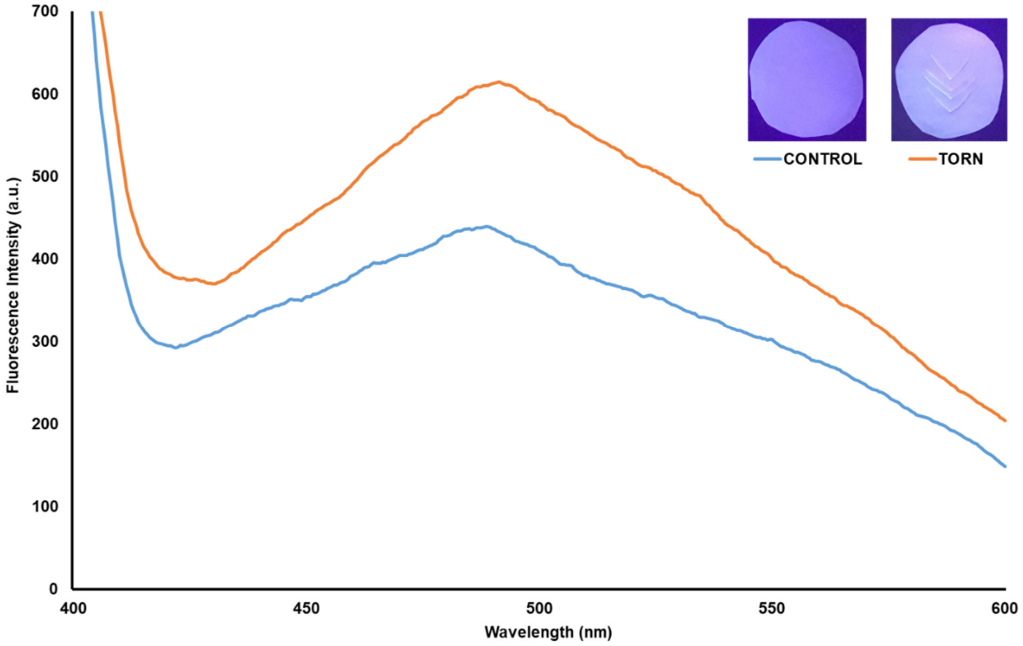

Figure S9. Fluorescence emission spectrum from **LC9** sample. (inset: NRL-Cur films under UV lamp at 380 nm)

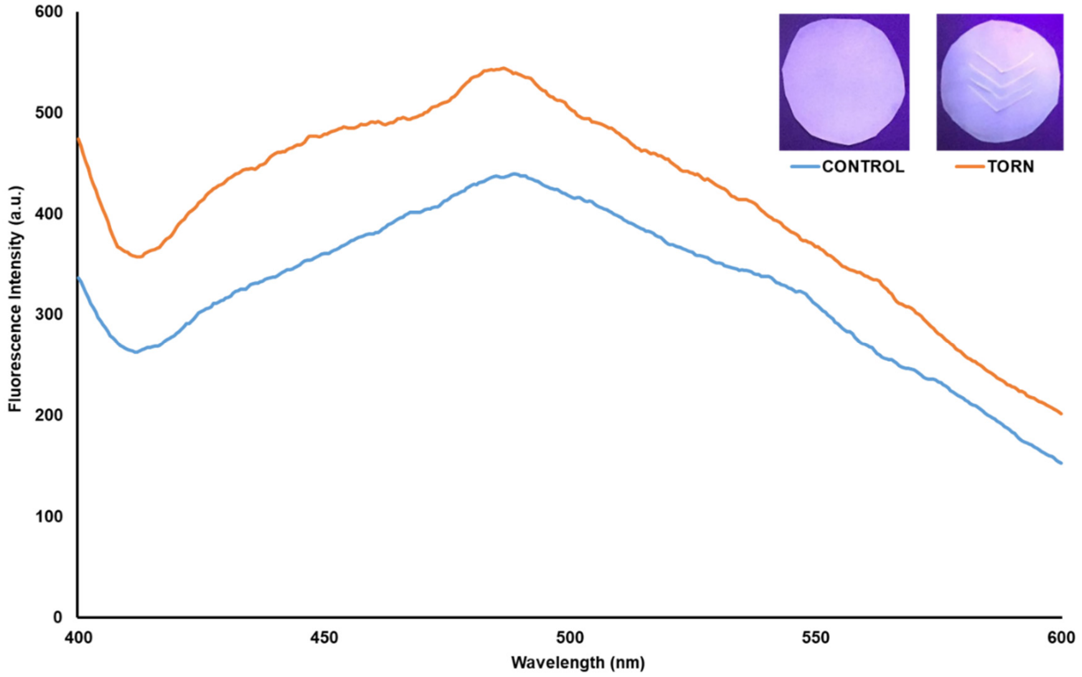

Figure S10. Fluorescence emission spectrum from **LC10** sample. (inset: NRL-Cur films under UV lamp at 380 nm)
